# The role of muscle depletion and visceral adiposity in HCC patients aged 65 and over undergoing TACE

**DOI:** 10.1186/s12885-021-08905-2

**Published:** 2021-10-30

**Authors:** Jihye Lim, Kyung Won Kim, Yousun Ko, Il-Young Jang, Yung Sang Lee, Young-Hwa Chung, Han Chu Lee, Young-Suk Lim, Kang Mo Kim, Ju Hyun Shim, Jonggi Choi, Danbi Lee

**Affiliations:** 1 Department of Gastroenterology, Asan Medical Center, University of Ulsan College of Medicine, 88, Olympic-ro 44-gil, Songpa-gu, Seoul, 05505 Republic of Korea; 2 Department of Radiology and Research Institute of Radiology, Asan Medical Center, University of Ulsan College of Medicine, Seoul, Republic of Korea; 3 Biomedical Research Center, Asan Institute for Life Sciences, Asan Medical Center, Seoul, Republic of Korea; 4 Division of Geriatrics, Department of Internal Medicine, Asan Medical Center, University of Ulsan College of Medicine, Seoul, Republic of Korea; 5 Asan Liver Center, Asan Medical Center, University of Ulsan College of Medicine, Seoul, Republic of Korea

**Keywords:** Carcinoma, hepatocellular, Chemoembolization, therapeutic, Muscle, skeletal, Intra-abdominal fat, Geriatrics, Life expectancy, Body mass index

## Abstract

**Background:**

The incidence of hepatocellular carcinoma (HCC) has been increasing among the elderly populations. Trans-arterial chemoembolization (TACE), a widely used first-line non-curative therapy for HCCs is an issue in geriatrics. We investigated the prognosis of elderly HCC patients treated with TACE and determined the factors that affect the overall survival.

**Methods:**

We included 266 patients who were older than 65 years and had received TACE as initial treatment for HCC. We analyzed the skeletal muscle index (SMI) and visceral-to-subcutaneous fat ratio (VSR) around the third lumbar vertebrae using computed tomography scans. Muscle depletion with visceral adiposity (MDVA) was defined by falling below the median SMI and above the median VSR value sex-specifically. We evaluated the overall survival in association with MDVA and other clinical factors.

**Results:**

The mean age was 69.9 ± 4.5 years, and 70.3% of the patients were men. According to the Barcelona Clinic Liver Cancer (BCLC) staging system, 29, 136, and 101 patients were classified as BCLC 0, A, and B stages, respectively, and 79 (29.7%) had MDVA. During the median follow-up of 4.1 years, patients with MDVA had a shorter life expectancy than those without MDVA (*P* = 0.007) even though MDVA group had a higher objective response rate after the first TACE (82.3% vs. 75.9%, *P* = 0.035). Multivariate analysis revealed that MDVA (Hazard ratio [HR] 1.515) age (HR 1.057), liver function (HR 1.078), tumor size (HR 1.083), serum albumin level (HR 0.523), platelet count (HR 0.996), tumor stage (stage A, HR 1.711; stage B, HR 2.003), and treatment response after the first TACE treatment (HR 0.680) were associated with overall survival.

**Conclusions:**

MDVA is a critical prognostic factor for predicting survival in the elderly patients with HCC who have undergone TACE.

**Supplementary Information:**

The online version contains supplementary material available at 10.1186/s12885-021-08905-2.

## Background

Along with increased socio-economic development and improvement in medical care, the number of aged patients newly diagnosed with hepatocellular carcinoma (HCC) has been increasing [1, 2]. However, there is limited data regarding anti-HCC treatments for the elderly population and available evidence suggests that widely used Eastern Cooperative Oncology Group Performance Status or Karnofsky Performance Status are insufficient predictors for elderly cancer patients [3, 4].

Trans-arterial chemoembolization (TACE), which involves injecting chemotherapeutic agents with embolic material to vessels that contribute to HCC, is the most frequently used treatment for HCC in real-world practice [5]. The indication of TACE is broad. TACE is the standard therapy for patients in the intermediate stage of HCC [6, 7], whereas for patients with early-stage HCC in whom surgery or local ablation is not feasible and in patients with advanced-stage HCC, TACE is an alternative [8]. TACE techniques are diverse according to the interventional methods and chemotherapeutic agents used; accordingly, TACE is classified as 1) conventional TACE, 2) drug-eluting bead TACE (DEB-TACE), wherein chemotherapeutic agents mixed with beads embolize the feeding vessel and release the drug slowly, and 3) trans-arterial radioembolization (TARE), in which microspheres loaded with a radioisotope are delivered to the target tumor [6]. The response to TACE is often unpredictable. For example, the median survival of intermediate stage HCC patients is estimated to be 2.5 years but, it is quite different from 5 to 25 months according to the liver function, number and size of tumors, or performance status [9]. TACE can sometimes cause serious complications, such as hepatic failure, duodenal perforation, pulmonary embolism, bile duct complications, acute renal failure, leukopenia, and even death, and the effects vary from person to person [10, 11].

The importance of body composition has been emphasized for HCC patients undergoing liver resection, transplantation, and systemic chemotherapy as proper body composition improves treatment tolerance [12–15]. Reduced muscle mass decreases functional capacity, worsens the quality of life, and eventually results in poor clinical outcomes [14]. Increased visceral adiposity is associated with insulin resistance and inflammation-promoting carcinogenesis [15]. However, the prognostic role of body composition for TACE, particularly in the aged population, is controversial [16–18].

It is often challenging for clinicians to make treatment plans, considering the relatively shorter life expectancy of older patients and the high possibility of serious adverse events because evidence regarding the safety and efficacy of TACE for older HCC patients is limited. Therefore, in this study, we aimed to investigate the factors affecting the survival of HCC patients over 65 years of age who were treated with TACE, with special focus on their body composition, as an objective health status evaluation method.

## Methods

### Patients

In this retrospective study, we screened the data of 649 HCC patients, aged 65 years or older, who were treated at the Asan Medical Center, Seoul, Republic of Korea, between January 2007 and December 2015, and followed up until December 2020. We defined “patients older than 65 years” as elderly because long-term care insurance is applicable from the age of 65 years in Korea [19]. All of these patients were treatment naïve. Among these silent HCC patients, we excluded the following: 1) 334 who were initially treated with interventions other than TACE (177 patients underwent hepatectomy; 134 patients, locoregional therapy; 13 patients, supportive care; 7 patients, systemic chemotherapy; and 3 patients, liver transplantation) and 2) 49 patients who were classified as having Barcelona Clinic Liver Cancer (BCLC) stage C, advanced HCC, at the time of diagnosis. Finally, we enrolled 266 patients initially treated with TACE for HCC (Supplementary Fig. 1). The diagnosis of HCC was based on typical contrast-enhanced imaging criteria or histopathological confirmation according to global practice guidelines [6, 7]. All data were anonymized and obtained from the electronic medical records of the Asan Medical Center. This study was approved by the Institutional Review Board of the Asan Medical Center, Seoul, Republic of Korea (2019–0986).

### Definitions of body composition

**Fig. 1:**
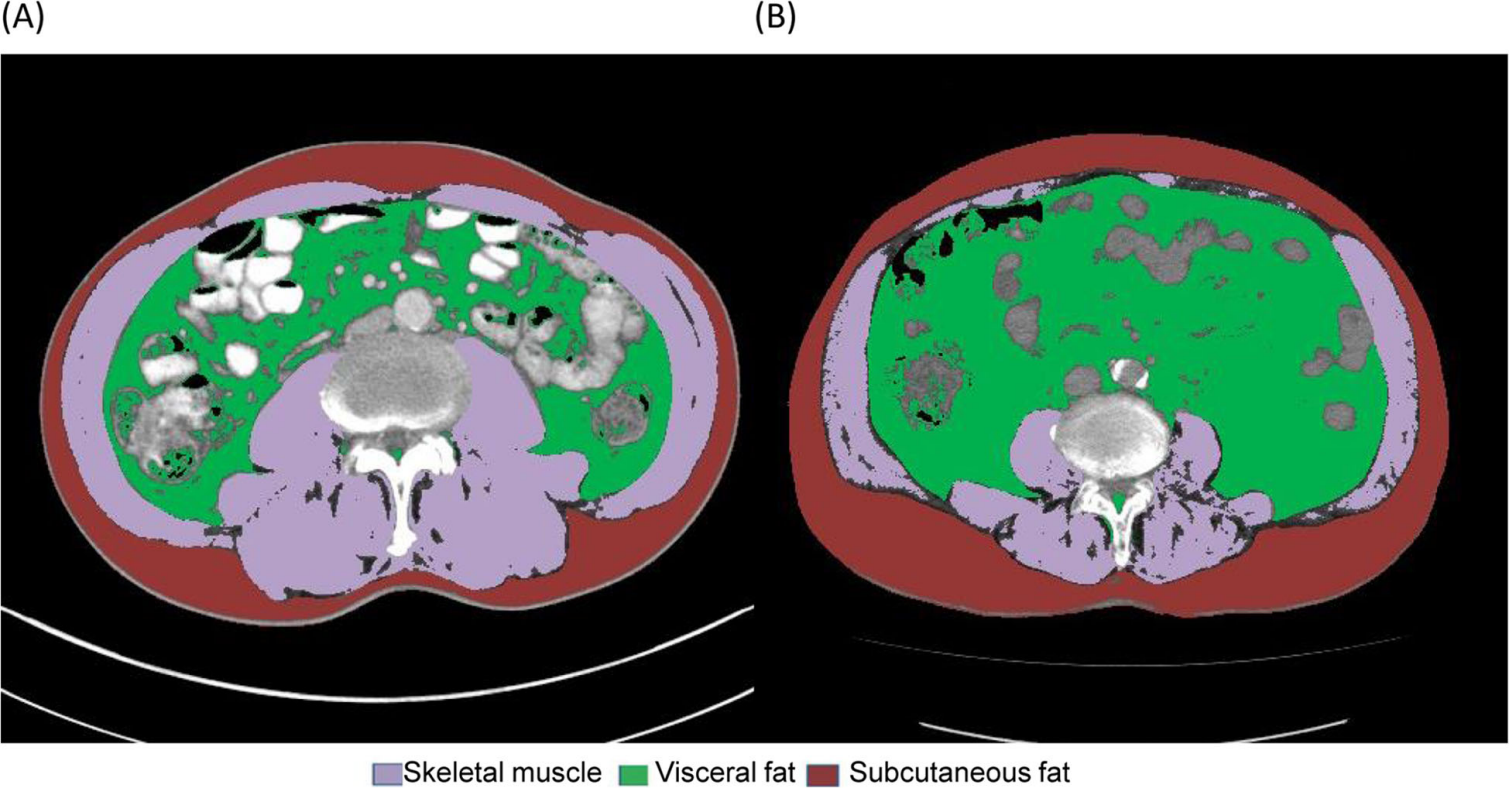
Cross-sectional computed tomography of two patients, body composition evaluated at the third lumbar vertebral level. Two patients with similar BMI values (24.4 vs. 24.8 kg/m^2^); one (A) had more skeletal muscle and less visceral adiposity than the other (B) (SMI, 63.7 vs.42.9 cm^2^/m^2^; VSR, 1.01 vs. 1.83). BMI, body mass index; SMI, skeletal muscle index; VSR, visceral-to-subcutaneous fat ratio

We analyzed cross-sectional computed tomography (CT) images at the third lumbar vertebra using AsanJ-Morphometry™ software to measure muscle and fat surface areas. The total abdominal muscle area included major large muscles and functional muscles, including the psoas, paraspinal, and abdominal wall muscles. We discriminated tissue using Hounsfield unit (HU) thresholds: muscle as − 29 to + 150 HU and fat as − 190 to − 30 HU (Fig. 1) [20]. The skeletal muscle index (SMI) was calculated as the total abdominal muscle area divided by the height squared in meters (cm^2^/m^2^). The visceral adipose tissue index and subcutaneous adipose tissue index were defined as the adipose tissue areas divided by the height squared in meters (cm^2^/m^2^). Muscle depletion was defined as a sex-specific SMI value less than the median value for all participants (as body composition differs greatly between the sexes). The visceral-to-subcutaneous fat ratio (VSR) was the ratio of the visceral fat area to the subcutaneous fat area. Visceral adiposity was defined as a sex-specific VSR greater than the median. Muscle depletion with visceral adiposity (MDVA) was defined as the presence of both muscle depletion and visceral adiposity. Patients were also grouped according to the body mass index (BMI). Sex-specific BMI values above the median were classified as the high BMI group and those below the median as the low BMI group.

### Clinicopathological variables

The following variables were examined as clinical and pathological predictors of prognosis: 1) patient-related factors, including age; sex; and history of diabetes, hypertension, cardiovascular incidence, other malignancy, renal failure needing dialysis, alcohol consumption, and smoking; 2) underlying liver disease-related factors, including laboratory data, ascites, variceal bleeding, and the model for end-stage liver disease (MELD) score; 3) tumor-related factors, including the number of tumors, maximal tumor size, serum alpha-fetoprotein level, infiltrative type of HCC, BCLC stage, and treatment response after the first TACE; and 4) body composition-related factors, including BMI, SMI, VSR, and the average HU of muscular and adipose tissue. Treatment response was evaluated via CT or magnetic resonance imaging 4 to 8 weeks after the first TACE according to the modified response evaluation criteria in solid tumors [21]. The objective tumor response rate (ORR) refers to the ratio of patients who have a complete or partial response [22]. To minimize inter-operator variability and enhance concordance among experts, the treatment response after TACE was evaluated by two hepatologists and two radiologists who have 7 to 20 years of experience [23].

### Statistical analysis

Statistical analyses were performed using IBM SPSS Statistic for Windows, version 21 (IBM Corp., Armonk, NY, USA). Baseline characteristics are presented as proportions for categorical variables and the mean ± standard deviations for continuous variables. Chi-squared analysis was used for categorical variables, and t-tests were used for continuous variables. The end date was the date of death or the date of the last follow-up. Hazard ratios (HRs) and 95% confidential intervals (CIs) for overall survival were estimated using Cox regression analysis. Variables with probability thresholds (*P*) < 0.05 from the univariate analysis were included in multivariate regression models and backward elimination. A multivariate *P* value less than 0.05 was considered statistically significant. Kaplan–Meier curves and log-rank testing were used to analyze the overall survival data.

## Results

### Characteristics of study participants

The main demographic and clinical data are shown in Table 1. Of the 266 patients, 187 were male, and the mean age was 69.9 ± 4.5 years. The most common cause of HCC was hepatitis B virus (HBV, 58.3%), and the MELD score was 8.4 ± 2.3. Half of the patients (54.8%) had multiple HCCs at the time of diagnosis, and the mean of maximal HCC tumor size was 3.7 ± 2.8 cm. Most patients (96.6%) were treated with conventional TACE. Among 266 patients, 29 (11%), 136 (51%), and 101 (38%) were classified as BCLC stage 0 (very early), A (early stage), and B (indeterminate state), respectively. Body compositions according to sex are presented in Supplementary Table 1. The median for SMI were 49.6 and 43.1 for men and women, respectively, and 0.34 and 0.22 for VSR for men and women, respectively. Patients with MDVA had larger tumors than patients without MDVA (4.3 ± 3.8 vs. 3.4 ± 2.3 cm, respectively; *P* = 0.046). However, there were no other clinicopathological differences between the two groups.

**Table 1:** Characteristics of the enrolled HCC patients

Variables	Total	Non-MDVA	MDVA	***P*** value
	(*N* = 266)	(*n* = 187)	(*n* = 79)	
Sex				0.991
Male	187 (70.3%)	132 (70.6%)	55 (69.6%)	
Female	79 (29.7%)	55 (29.4%)	24 (30.4%)	
Age (years)	69.9 ± 4.5	69.6 ± 4.3	70.8 ± 4.9	0.051
BMI (kg/m^2^)	24.4 ± 3.5	25.6 ± 3.2	21.7 ± 2.5	< 0.001
Diabetes	77 (28.9%)	60 (32.1%)	17 (21.5%)	0.112
Hypertension	111 (41.7%)	78 (41.7%)	33 (41.8%)	> 0.999
Cardiovascular attack	10 (3.8%)	8 (4.3%)	2 (2.5%)	0.740
Other malignancy	17 (6.4%)	11 (5.9%)	6 (7.6%)	0.805
Renal failure on dialysis	9 (3.4%)	7 (3.7%)	2 (2.5%)	0.898
Alcohol	146 (54.9%)	103 (55.1%)	43 (54.4%)	> 0.999
Smoking	128 (48.1%)	89 (47.6%)	39 (49.4%)	0.896
Etiology				0.098
Hepatitis B	155 (58.3%)	116 (62.0%)	39 (49.4%)	
Hepatitis C	60 (22.6%)	36 (19.3%)	24 (30.4%)	
Others	51 (19.2%)	35 (18.7%)	16 (20.3%)	
Variceal bleeding	2 (0.8%)	2 (1.1%)	0 (0.0%)	0.884
Ascites	14 (5.3%)	8 (4.3%)	6 (7.6%)	0.420
MELD score	8.4 ± 2.3	8.4 ± 2.1	8.3 ± 2.7	0.826
Platelet (×10^3^/uL)	124.1 ± 58.7	123.6 ± 56.7	125.4 ± 63.4	0.828
Prothrombin time (INR)	1.11 ± 0.13	1.12 ± 0.11	1.11 ± 0.16	0.675
Creatinine (mg/dl)	0.9 ± 0.7	0.9 ± 0.6	1.0 ± 1.0	0.503
AST (IU/L)	48.5 ± 33.0	49.0 ± 30.2	47.3 ± 39.1	0.731
ALT (IU/L)	41.9 ± 30.1	40.3 ± 24.8	45.5 ± 39.8	0.285
Albumin (g/dl)	3.5 ± 0.5	3.5 ± 0.5	3.5 ± 0.5	0.777
Bilirubin (mg/dl)	1.0 ± 0.5	1.0 ± 0.5	0.9 ± 0.4	0.815
Number of tumors				0.943
Single	122 (45.9%)	85 (45.5%)	37 (46.8%)	
Multiple	144 (54.1%)	102 (54.5%)	42 (53.2%)	
Size of tumors (cm)	3.7 ± 2.8	3.4 ± 2.3	4.3 ± 3.8	0.046
Infiltrative type of HCC	3 (1.1%)	3 (1.6%)	0 (0.0%)	0.619
BCLC				0.327
stage 0	29 (10.9%)	20 (10.7%)	9 (11.4%)	
stage A	136 (51.1%)	101 (54.0%)	35 (44.3%)	
stage B	101 (38.0%)	66 (35.3%)	35 (44.3%)	
Serum AFP (ng/mL)	376.5 ± 1308.9	369.2 ± 1382.4	393.6 ± 1123.9	0.881
TACE method				0.175
Conventional TACE	257 (96.6%)	183 (97.9%)	74 (93.7%)	
DEB-TACE	9 (3.4%)	4 (2.1%)	5 (6.3%)	

Values are expressed as the mean ± standard deviation, or frequency (%)

AFP, alpha-fetoprotein; AST, aspartate transaminase; BCLC, Barcelona Clinic Liver Cancer; BMI, body mass index; HCC, hepatocellular carcinoma; INR, international normalized ratio; MDVA, muscle depletion with visceral adiposity; MELD, model for end-stage liver disease; TACE, trans-arterial chemoembolization; DEB-TACE, drug eluting bead TACE

### Treatment response after TACE and overall survival

The treatment responses were evaluated by CT or MRI after the first TACE (median 46 days), and the results were shown in Table 2. The overall response rate (complete response and partial response) of the entire cohort was 77.8, and 75.9% and 82.3% in non-MDVA and MDVA groups, respectively (*P* = 0.035). Four patients could not undergo evaluation; one patient with MDVA diad 10 days after TACE, and 3 patients (2 with MDVA, and 1 without MDVA) were unable to get further evaluation nor anti-cancer treatment because of poor general condition.

**Table 2:** Objective response rate and disease control rate of the entire cohort

Variables	Total (*N* = 266)	Non-MDVA (*n* = 187)	MDVA (n = 79)	P value
Best response				0.017
Complete response	106 (39.8%)	78 (41.7%)	28 (35.4%)	
Partial response	101 (38%)	64 (34.2%)	37 (46.8%)	
Stable disease	42 (15.8%)	36 (19.3%)	6 (7.6%)	
Progressive disease	13 (4.9%)	8 (4.3%)	5 (6.3%)	
Not evaluable	4 (1.5%)	1 (0.5%)	3 (3.8%)	
Objective response rate	207 (77.8%)	142 (75.9%)	65 (82.3%)	0.035

Values are expressed as frequency (%)

MDVA, muscle depletion with visceral adiposity

**Fig. 2:**
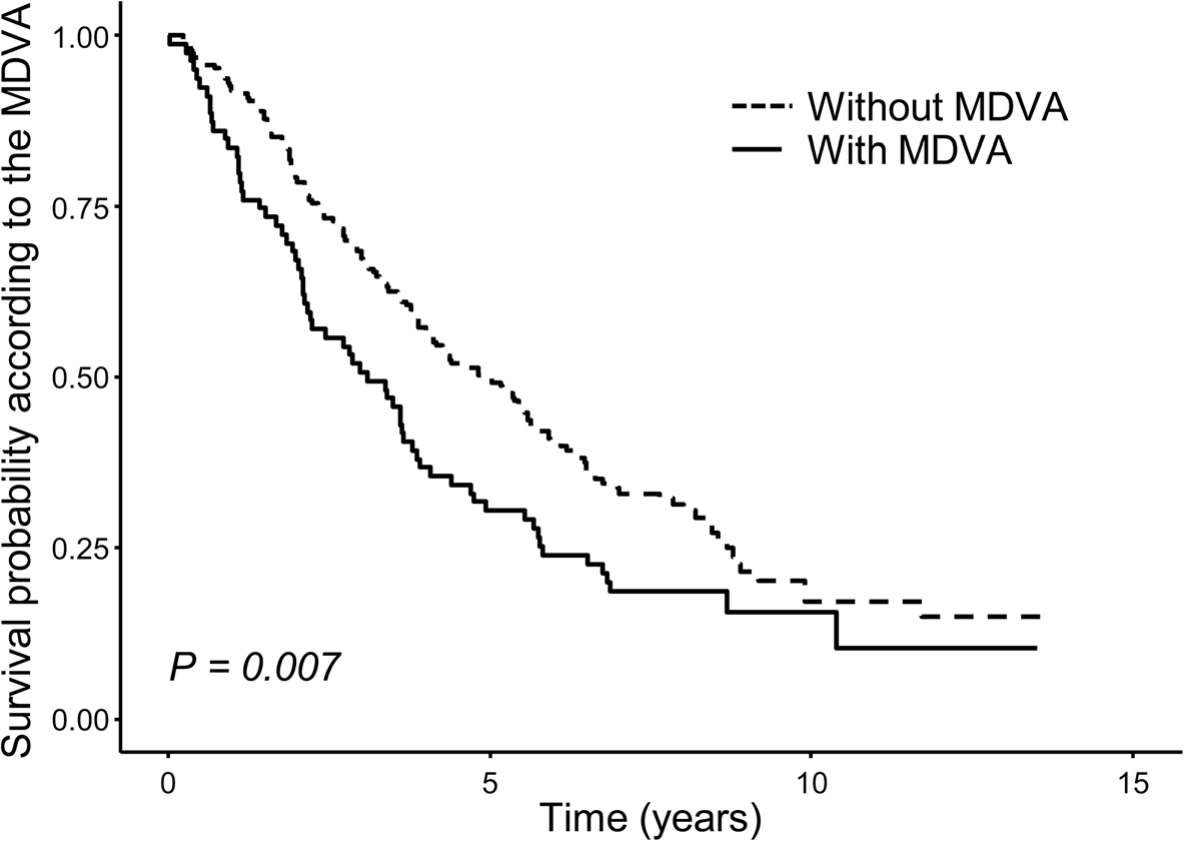
Kaplan-Meier analysis for survival in geriatric HCC patients treated with TACE according to the MDVA. Patients without MDVA had a better survival rate. HCC, hepatocellular carcinoma; MDVA, muscle depletion with visceral adiposity; TACE, trans-arterial chemoembolization

During the median follow-up of 4.1 years (range, 2.0–6.8 years), 76.7% were dead, among which 73.8 and 83.5% were from the non-MDVA and MDVA groups (*P* = 0.119). The patients with MDVA had a shorter life expectancy than those without MDVA (83.5% vs. 91.4% at 1 year; 30.4% vs. 49.7% at 5 years, respectively; *P* = 0.007; Fig. 2). The first quartiles of both SMI and VSR groups were compared with others, and the relationship with body composition and overall survival showed significant difference (*P* = 0.041; Supplementary Fig. 2). Meanwhile, BMI was not related to long-term survival (*P* = 0.570; Supplementary Fig. 3).

The presence of MDVA (HR 1.501, *P* = 0.007) and the following variables were significantly associated with poor survival in the univariate analysis; increased age (HR 1.060, *P* < 0.001), underlying cause of liver disease (HCV, HR 1.802; other than HBV and HCV, HR 1.314, *P* = 0.002); presence of ascites (HR 1.910, *P* = 0.030); higher MELD score (HR 1.100, *P* < 0.001); larger size of the tumor (HR 1.060, *P* = 0.006); lower serum albumin level (HR 0.467, *P* < 0.001); lower platelet count (HR 0.996, *P* = 0.004); higher BCLC grade (stage A, HR 1.691; stage B, HR 2.062; *P =* 0.022); and poor response after first TACE (objective tumor response HR 0.614, *P* = 0.003) (Table 3). A further multivariate model revealed that the presence of MDVA was an independent predictor for the survival of elderly patients with HCC after TACE (HR 1.515, *P* = 0.009). In addition, increased age (HR 1.057, *P* < 0.001), higher MELD score (HR 1.078, *P* = 0.027), bigger tumor size (HR 1.083, *P* = 0.004), lower serum albumin level (HR 0.523, *P* < 0.001), lower platelet level (HR 0.996, *P* = 0.006), more advanced BCLC stage (stage A, HR 1.711; stage B, HR 2.003; *P =* 0.015), not obtaining objective response (HR 0.680, *P* = 0.023) were significant prognostic factors for short life expectancy (Table 3).

**Table 3:** Univariate and multivariate analyses for overall survival of the entire cohort

	Univariate analysis		Multivariate analysis	
	HR (95% CI)	***P*** value	HR (95% CI)	***P*** value
Age (year)	1.060 (1.030–1.090)	< 0.001	1.057 (0.946–1.024)	< 0.001
Sex	0.982 (0.726–1.330)	0.905		
BMI (kg/m^2^)	0.983 (0.942–1.030)	0.446		
Diabetes	1.170 (0.871–1.570)	0.295		
Hypertension	0.759 (0.573–1.010)	0.056		
Cardiovascular attack	1.330 (0.703–2.510)	0.381		
Other malignancy	1.400 (0.810–2.410)	0.229		
Renal failure on dialysis	1.920 (0.984–3.760)	0.056		
Alcohol	0.937 (0.711–1.240)	0.645		
Smoking	0.922 (0.700–1.220)	0.565		
Etiology		0.002		
Hepatitis B	1	–		
Hepatitis C	1.802 (1.295–2.507)	< 0.001		
Others	1.314 (0.917–1.883)	0.137		
Variceal bleeding	1.760 (0.437–7.110)	0.426		
Ascites	1.910 (1.060–3.440)	0.030		
MELD score	1.100 (1.050–1.160)	< 0.001	1.078 (0.928–1.009)	0.027
Number of tumors	1.250 (0.947–1.650)	0.116		
Size of tumor (cm)	1.060 (1.020–1.110)	0.006	1.083 (0.923–1.026)	0.004
Albumin (g/dL)	0.467 (0.358–0.611)	< 0.001	0.523 (1.912–0.383)	< 0.001
Platelet (uL)	0.996 (0.993–0.999)	0.004	0.996 (1.004–0.993)	0.006
BCLC		0.022		0.015
stage 0	1	–	1	–
stage A	1.691 (1.012–2.824)	0.045	1.711 (0.585–1.012)	0.045
stage B	2.062 (1.221–3.480)	0.007	2.003 (0.499–1.148)	0.014
Infiltrative type of HCC	1.210 (0.300–4.870)	0.791		
Serum AFP (ng/mL)	1.000 (1.000–1.000)	0.573		
MDVA	1.501 (1.118–2.014)	0.007	1.515 (0.660–1.112)	0.009
Objective tumor response	0.614 (0.448–0.843)	0.003	0.680 (1.471–0.488)	0.023

AFP, alpha-fetoprotein; BCLC, Barcelona Clinic Liver Cancer; BMI, body mass index; HCC, hepatocellular carcinoma; MDVA, muscle depletion with visceral adiposity; MELD, model for end-stage liver disease

### Subgroup analysis of overall survival according to the BCLC stages

**Fig. 3:**
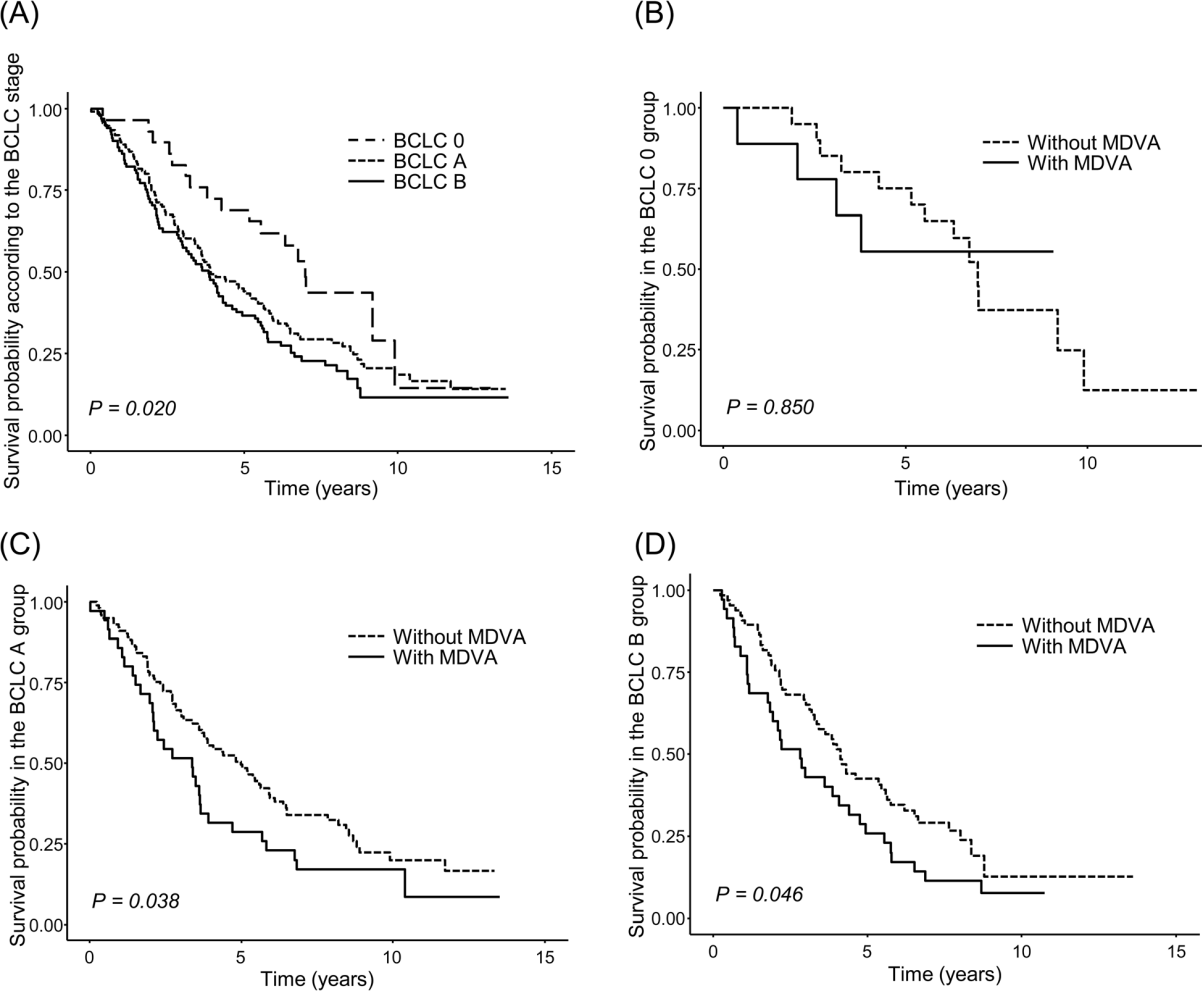
Kaplan-Meier analysis for survival in geriatric HCC patients. Kaplan-Meier analysis for survival in geriatric HCC patients treated with TACE (A) according to the BCLC and that according to MDVA in each stage; (B) stage 0, (C) stage A, and (D) stage B. Life expectancy was different depending on the cancer stage; patients with BCLC stage 0 had the longest survival expectancy. In BCLC stages A and B, MDVA was important for survival. BCLC, Barcelona Clinic Liver Cancer; HCC, hepatocellular carcinoma; MDVA, muscle depletion with visceral adiposity; TACE, trans-arterial chemoembolization.

Patients with BCLC stage 0 had the longest expected survival compared with those with stage A or stage B (stage 0 vs. stage A vs. stage B; 96.6% vs. 89.7% vs. 87.1% at 1 year; 69.0% vs. 44.1% vs. 36.6% at 5 years, respectively; *P* = 0.020) (Fig. 3).

As shown in Fig. 3, there was no significant survival difference between MDVA and non-MDVA group in BCLC stage 0; but patients with MDVA had shorter life expectancy than non-MDVA patients in BCLC stage A and B, respectively. In BCLC stage A, the survival rates according to the presence of MDVA were 91.1% vs. 85.7% at 1 year, and 49.5% vs. 28.6% at 5 years in non-MDVA vs. MDVA patients (*P* = 0.038). Likewise, in BCLC stage B, non-MDVA patients showed higher survival rates (90.9% vs. 80.0% at 1 year, 42.4% vs. 25.7% at 6 years, respectively; *P* = 0.046). The univariate analysis also showed that MDVA was associated with survival in BCLC stage A (HR 1.562, *P* = 0.040) and B (HR 1.566, *P* = 0.048). When early to intermediate stage of HCC (BCLC stage A and B) patients are considered, those with MDVA tended to have a poorer outcome than those without MDVA even for the different cancer stages; patients with BCLC stage A with MDVA had shorter life expectancy than those with BCLC stage B without MDVA (Supplementary Fig. 4).

## Discussion

We investigated the factors that affect the overall survival for newly diagnosed older HCC patients treated with TACE. We demonstrated that chronological age, poor liver function with higher MELD score, tumor size, lower albumin and platelet level, higher cancer stage, non-response to the TACE, as well as MDVA were major risk factors for poor survival outcomes. The number of older HCC patients is on the rise, but physicians have a hard time assessing the frailty or geriatric conditions of these patients. Our findings, especially MDVA, offer a more objective assessment paradigm.

TACE is a well-established treatment, but its adverse events have always been a concern for older patients as they are more vulnerable to external stresses [6, 24–26]. Therefore, TACE should be reserved for select patients, and the treatment should be performed using a customized approach based on the tumor stage, liver function, and health condition [27]. Simple observation of body weight or BMI in older patients have been used to evaluate the general condition; however, they are not enough to reflect the actual body composition, as patients with similar BMI do not necessarily have similar body composition. In our study, we did not find a relationship between BMI and survival probability.

In our study, MDVA was found to be an important prognostic factor in older HCC patients treated with TACE. Muscle depletion and visceral adiposity are important predictors of overall health and clinical outcomes for various diseases [14, 28, 29]. In terms of malignancies, the diminished muscle mass makes patients more vulnerable to chemotherapy toxicities [30, 31]. Liver disease and muscle wasting impact one another. Liver cirrhosis accelerates muscle wasting by altering the processes of protein turnover and energy disposal, leading to metabolic changes. It impairs immune responses, aggravates ascites, and worsens the quality of life [32–34]. Adipose tissue distribution is influenced by sex, age, race, diet, and physical activity levels [35, 36]. Visceral adiposity is known to be distinctly associated with metabolic syndrome, risk of cancers, and mortality [1, 37, 38]. This is because visceral adipose tissue releases pro-inflammatory factors, such as adiponectin, tumor necrosis factor, interleukin 6, and free fatty acids [39]. These substances directly flow into the liver through the portal vein, and it causes liver inflammation, cirrhosis, and even HCC [29].

One of the interesting findings of our study was that the clinical significance of MDVA to survival was maintained in the entire cohort, in each early and intermediate stage. Further, the treatment response after the first TACE was better in the MDVA than in the non-MDVA group. The objective response has substantial importance for HCC patients undergoing TACE because it correlates well to overall survival [22]. In our study, even if MDVA patients had a higher objective response rate, their survival period was shorter than non-MDVA patients. This demonstrates the importance of MDVA in geriatrics with HCC undergoing TACE. MDVA is one of the parameters of frailty [40]. As frail patients are more vulnerable to stress and have diminished physiological reserve, they suffer more from treatment-related complications and are intolerant to chemotherapy than non-frail patients [41]. Indeed, 15.2% of MDVA patients did not undergo any further anti-cancer treatment after the first TACE, while only 5.3% of non-MDVA patients underwent supportive care. Impaired immune system owing to MDVA makes patients more susceptible to infection, which is the common cause of non-cancer-related death among HCC patients [42].

In clinical practice, the BCLC staging system is widely used for predicting outcomes; however, early-stage (BCLC A) patients treated with TACE have a slightly better or similar survival rate compared with intermediate stage (BCLC B) patients treated with TACE in other studies [6, 43–46]. It suggests that there might be crucial factors, other than the BCLC stage, that affect prognosis in early- or intermediate-stage HCC patients treated with TACE. Our cohort of elderly HCC patients showed that body composition, which comprehensively reflects the geriatric health status, might be one of the important prognostic factors for HCC patients treated with TACE [13]. It demonstrates that lifestyle modification to build up the muscle and reduce visceral fat would be helpful for aged HCC patients treated with TACE, in addition to the appropriate anti-cancer treatment. Healthy lifestyle modifications for cancer patients, such as resistance training exercise or aerobic exercise, proved their role for increasing muscle mass and decreasing visceral fat [47–49]. In our institution, we are trying to properly manage in-hospital patients with poor body conditions. For example, we have a nutrition support team comprising doctors, nurses, and nutritionists. The team screens malnourished in-hospital patients based on the BMI as well as rapid change of body weight and makes proper interventions such as intravenous nutritional supplements and tailored diets. We also have a rehabilitation and exercise program for bed-ridden patients. We’d like to broaden this program to outpatient clinics as we do not have a clinical program for outpatients yet.

The effect of body composition of HCC patients is suggested in many studies. Muscle depletion or visceral fat was related to the prognosis of not only TACE, but also of other treatments such as hepatectomy, liver transplantation, radiofrequecy ablation, radiotherapy, or systemic chemotherapy including sorafenib [50–54]. A study from Germany revealed that sarcopenia, defined as psoas muscle volume divided by height square, as well as rapid reduction of muscle volume were poor prognostic factors for 56 TACE-treated HCC patients. A previous American study suggested that visceral fat density appeared to predict the 1-year survival and hepatic decompensation after treatment for 75 HCC patients who underwent TACE, although the study included a heterogenous population including various ages, tumor stages, and liver function, which might affect the visceral fat [50]. In our study, we particularly focused on 266 intermediate stage HCC patients aged 65 years or over to reduce the body composition bias, inevitably caused by disease status or age [55]. Personalized treatments are essential to deal with elderly HCC patients as tailored medicine is more fundamental than ever. It has always been a challenge to identify high-risk patients with poor outcomes. Here, we revealed the importance of body composition for TACE-treated elderly HCC patients, which helps the hepatologists to make more effective management strategies against these types of patients with HCC. Simple evaluation for MDVA would be useful to establish tailored therapeutic and intervention plans for elderly HCC patients, as MDVA status was associated with poor survival.

This study has several limitations. First, MDVA was defined by the median value within our cohort, as there is no internationally accepted standardized value. Body composition is largely different within and among populations; hence, it would be inappropriate to apply our absolute values for other races or ethnicities [56]. Second, muscle function and power are as important as muscle mass; however, these factors were omitted from the analysis, as these details were unavailable for retrospective study design [57]. We plan to further investigate muscle function and power by measuring handgrip strength and walking speed. However, there might be a concern that MDVA at the diagnosis is affected by not only general physical status but also the malignancy. For our study, all patients were early- or intermediate-stage at the time of diagnosis and they had relatively preserved liver function; hence, body composition would be more strongly influenced by geriatric conditions. The long-term clinical courses of our patients were not evaluated in this study; however, we aimed to determine the prognostic factors for survival at the time of diagnosis.

In conclusion, our results underscore the clinical implication for body composition in the elderly HCC patients undergoing TACE. Body composition, especially muscle depletion and visceral adiposity, should be considered as an important prognostic parameter in clinical practice, and increased attention to these factors might lead to better clinical outcomes.

## Supplementary Information

**Additional file 1.**

## Data Availability

The raw data of the current study are not publicly available due to the protection of the participants’ personal information but are available from the corresponding author on reasonable request.

## Abbreviations

HCC: hepatocellular carcinoma
TACE: trans-arterial chemoembolization
SMI: skeletal muscle index
VSR: visceral-to-subcutaneous fat ratio
MDVA: muscle depletion with visceral adiposity
BCLC: Barcelona Clinic Liver Cancer
HU: hounsfield unit
MELD: model for end-stage liver disease
BMI: body mass index
HR: hazard ratio
CI: confidential interval
HBV: hepatitis B virus
