# Supplementary material for: The role of muscle depletion and visceral adiposity in HCC patients aged 65 and over undergoing TACE
Source: BMC Cancer. 2021 Oct 30;21:1164. doi: 10.1186/s12885-021-08905-2 (PMC8557070; doi:10.1186/s12885-021-08905-2)
Supplement: Supplementary file 1 — Additional file 1. [file 12885_2021_8905_MOESM1_ESM.docx]

**Supplementary Material**

**Clinical implications of the body composition among older hepatocellular carcinoma patients treated with trans-arterial chemoembolization**

Jihye Lim, Kyung Won Kim, Yousun Ko, Il-Young Jang, Yung Sang Lee, Young-Hwa Chung, Han Chu Lee, Young-Suk Lim, Kang Mo Kim, Ju Hyun Shim, Jonggi Choi, Danbi Lee

Table of contents

| **Supplementary Table 1.** | Body composition of the entire cohort according to sex |
| --- | --- |
| **Supplementary Figure 1.** | Study design and enrollment flowchart |
| **Supplementary Figure 2.** | Kaplan-Meier analysis for survival in geriatric HCC patients treated with TACE according to the first quartiles of both SMI and VSR |
| **Supplementary Figure 3.** | Kaplan-Meier analysis for survival in geriatric HCC patients treated with TACE according to the BMI |
| **Supplementary Figure 4.** | Kaplan-Meier analysis for survival in geriatric HCC patients treated with TACE in BCLC stage A and B |

**Supplementary Table 1**. Body composition of the entire cohort according to sex

Values are expressed as mean ± standard deviation.

BMI, body mass index; SATI, subcutaneous adipose tissue index; SMI, skeletal muscle index; VATI, visceral adipose tissue index; VSR, visceral to subcutaneous fat ratio

**Supplementary Figure 1**. Study design and enrollment flowchart


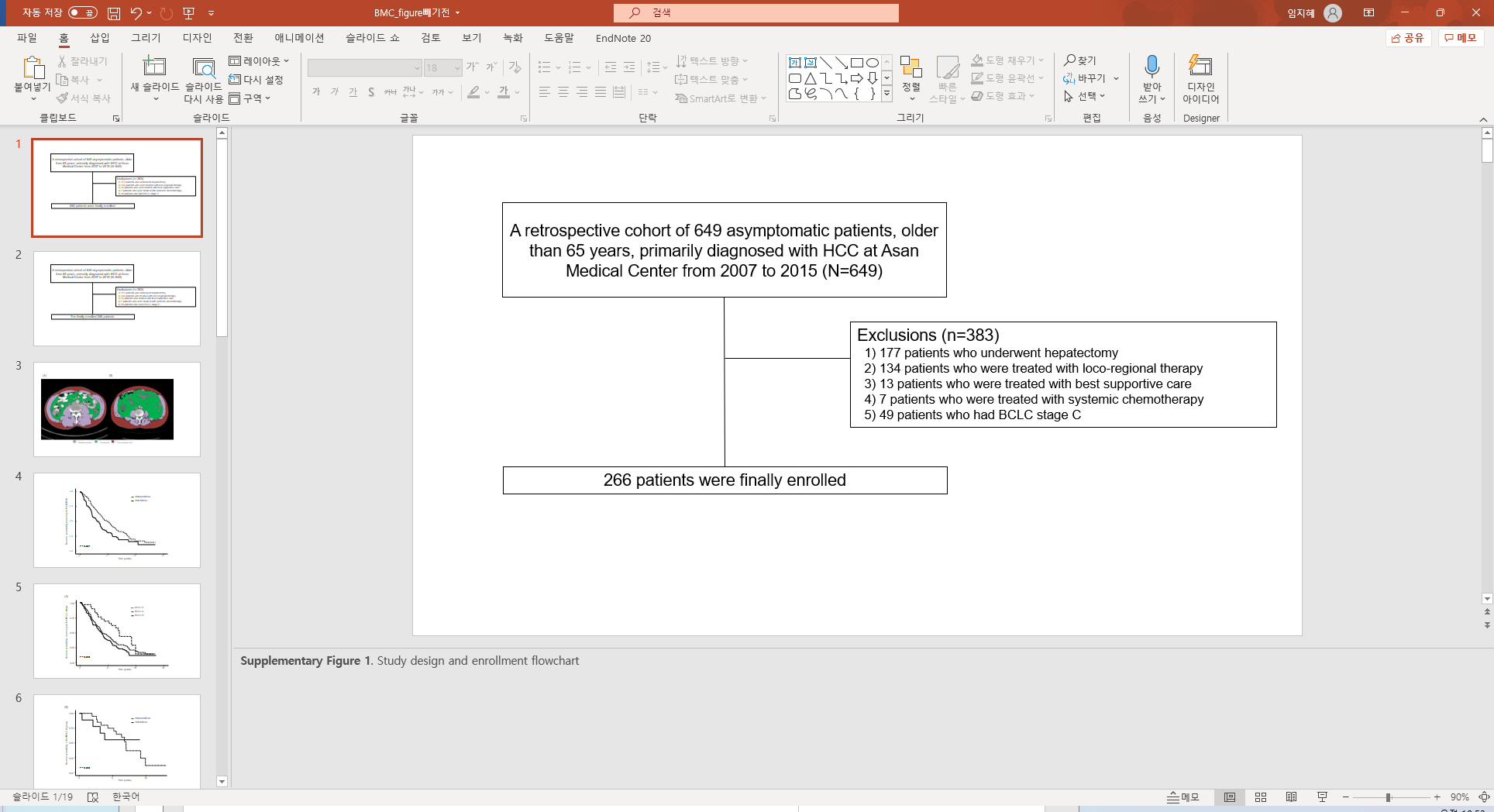


BCLC, Barcelona Clinic Liver Cancer; HCC, hepatocellular carcinoma; TACE, trans-arterial chemoembolization

**Supplementary Figure 2.** Kaplan-Meier analysis for survival in geriatric HCC patients treated with TACE according to the first quartiles of SMI and VSR
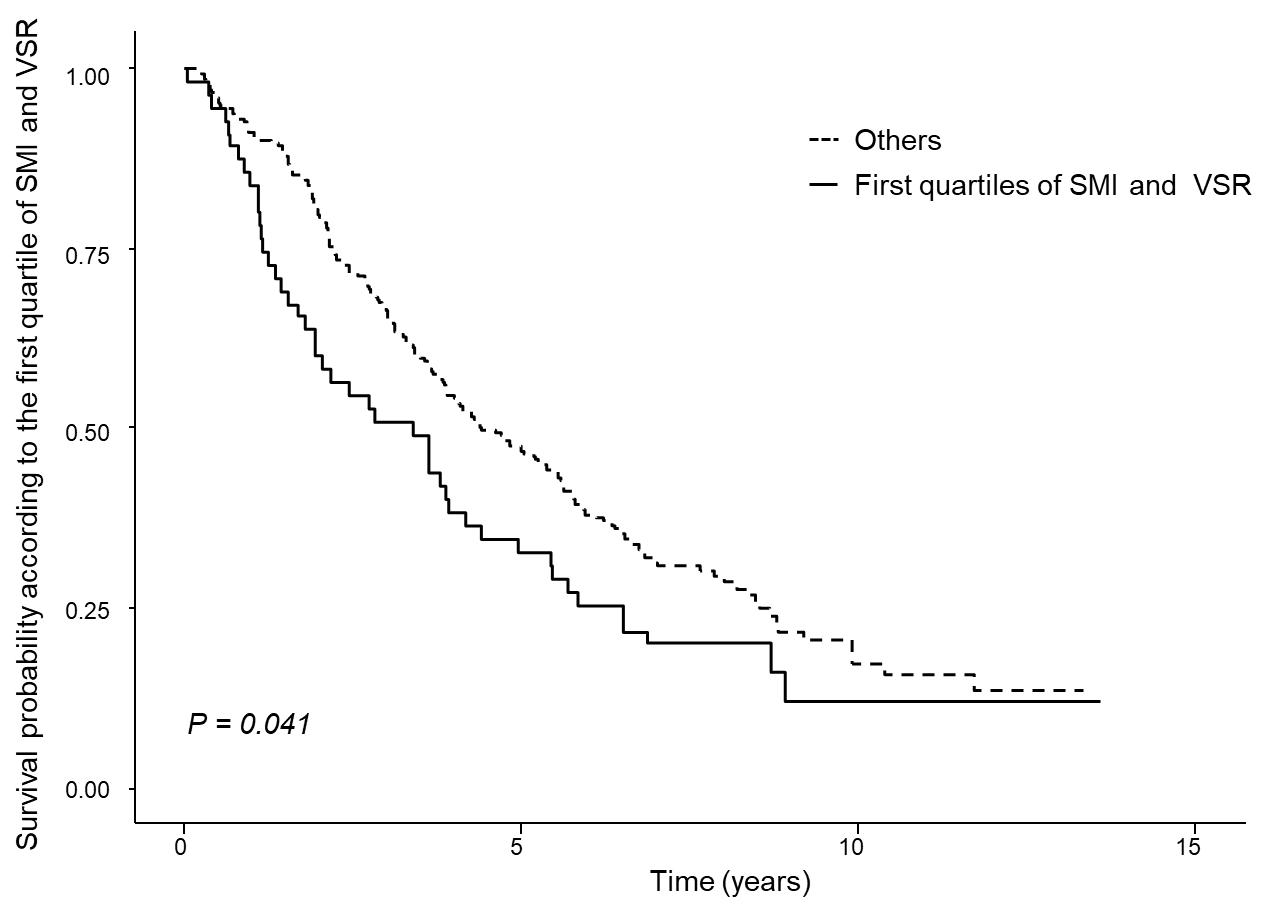
.

HCC, hepatocellular carcinoma; TACE, trans-arterial chemoembolization; SMI, skeletal muscle index; VSR, visceral to subcutaneous fat ratio

**Supplementary Figure 3.** Kaplan-Meier analysis for survival in geriatric HCC patients treated with TACE according to the BMI.


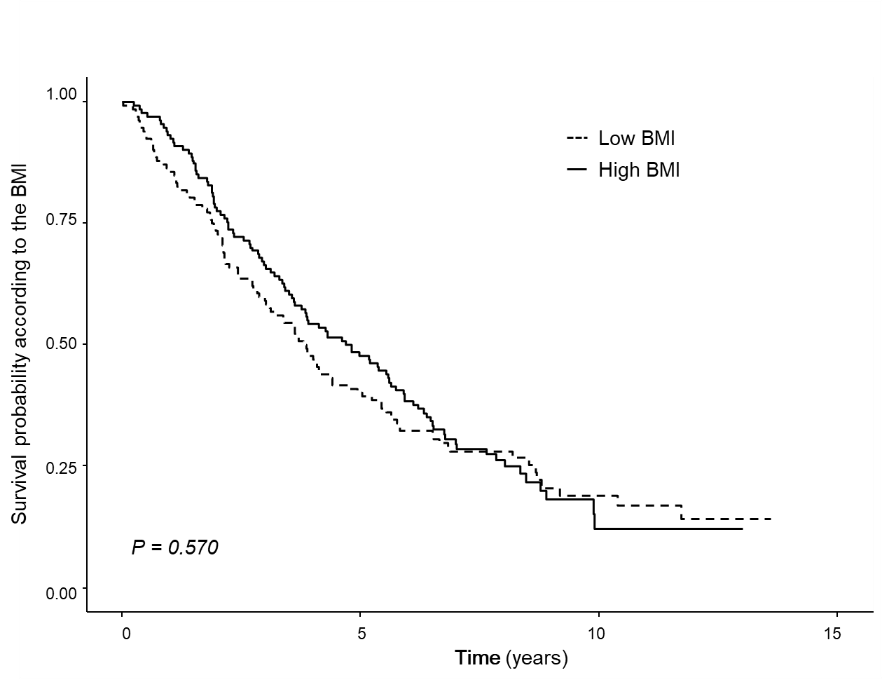


HCC, hepatocellular carcinoma; TACE, trans-arterial chemoembolization; BMI, body mass index

**Supplementary Figure 4**. Kaplan-Meier analysis for survival in geriatric HCC patients treated with TACE according to BCLC stages A and B.


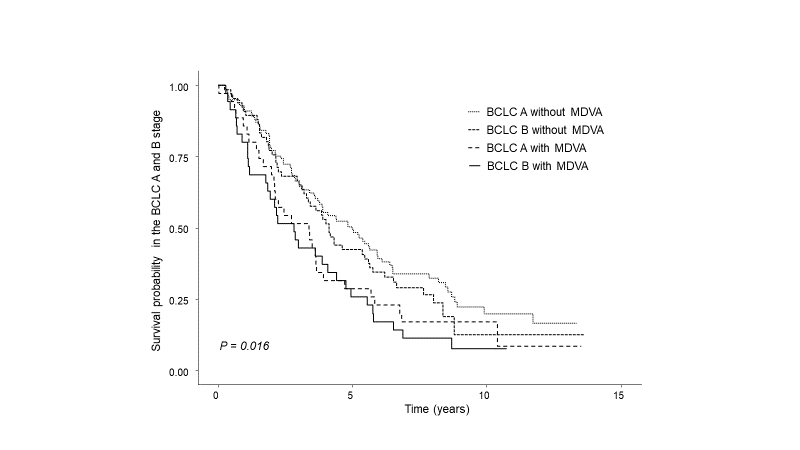


In the BCLC stage A or B group, MDVA was associated with overall survival, and BCLC stage B patients without MDVA tended to live longer than BCLC stage A patients with MDVA.

BCLC, Barcelona Clinic Liver Cancer; HCC, hepatocellular carcinoma; MDVA, muscle depletion with visceral adiposity; TACE, trans-arterial chemoembolization
